# CXCR6-CXCL16 Axis Promotes Breast Cancer by Inducing Oncogenic Signaling

**DOI:** 10.3390/cancers13143568

**Published:** 2021-07-16

**Authors:** Hina Mir, Neeraj Kapur, Dominique N. Gales, Praveen K. Sharma, Gabriela Oprea-Ilies, Anita T. Johnson, Rajesh Singh, Shailesh Singh

**Affiliations:** 1 Department of Microbiology, Biochemistry and Immunology, Morehouse School of Medicine, Atlanta, GA 30310, USA; Hmir@msm.edu (H.M.); neekap2008@gmail.com (N.K.); dncgales31@gmail.com (D.N.G.); rsingh@msm.edu (R.S.); 2 Cancer Health Equity Institute, Morehouse School of Medicine, Atlanta, GA 30310, USA; 3 Centre for Life Sciences, Central University of Jharkhand, Jharkhand 835205, India; pksharma@cuj.ac.in; 4 Department of Pathology & Laboratory Medicine, Winship Cancer Institute, Emory University School of Medicine, Atlanta, GA 30322, USA; goprea@emory.edu; 5 Comprehensive Cancer Care Network, Cancer Treatment Center of America, Atlanta, GA 30265, USA; anita.johnson2@ctca-hope.com; 6 Cell and Molecular Biology Program, Winship Cancer Institute, Emory University School of Medicine, Atlanta, GA 30322, USA

**Keywords:** breast cancer, chemokine, chemokine receptor, ADAM10

## Abstract

**Simple Summary:**

Breast cancer (BrCa) is the second leading cause of cancer-related deaths in American women, and its incidence is on the rise. Insufficient understanding of the mechanisms leading to BrCa limits the effectiveness of the treatment. In this article, we show the importance of a chemokine axis-CXCR6/CXCL16 in supporting BrCa progression. We have delineated BrCa-promoting mechanisms induced by this chemokine axis at the molecular level. This work projects the therapeutic significance of CXCR6/CXCL16 signaling for the treatment of BrCa.

**Abstract:**

Precise mechanisms underlying breast cancer (BrCa) metastasis are undefined, which becomes a challenge for effective treatments. Chemokine signaling instigates the trafficking of cancer cells in addition to leukocytes. This study aimed to ascertain the clinical and biological significance of the CXCR6/CXCL16 signaling axis in the pathobiology of BrCa. Our data show a higher expression of CXCR6 in BrCa cell lines and tissues. Stage-III BrCa tissues express significantly higher CXCR6 compared to stage-II tissues. The ligand, CXCL16, could remain tethered to the cell surface, and, after proteolytic shedding of the ectodomain, the N-terminal fragment is released, converting it to its oncogenic, soluble form. Like CXCR6, N-terminal CXCL16 and ADAM-10 were significantly higher in stage-III than stage-II, but no significant difference was observed in the C-terminal fragment of CXCL16. Further, stimulation of the CXCR6/CXCL16 axis activated Src, FAK, ERK1/2, and PI3K signaling pathways, as per antibody microarray analysis, which also underlie CXCL16-induced F-actin polymerization. The CXCR6/CXCL16 axis induces cytoskeleton rearrangement facilitating migration and invasion and supports BrCa cell survival by activating the PI3K/Akt pathway. This study highlights the significance of the CXCR6/CXCL16 axis and ADAM10 as potential therapeutic targets for advanced-stage BrCa.

## 1. Introduction

Breast cancer (BrCa) is the most common malignancy and the second leading cause of cancer-related deaths in American women [1,2]. Current predictions suggest that the worldwide incidence of BrCa and related death is on the rise [3]. Hormone therapy, surgery, chemotherapy, radiation, and targeted therapy are helpful; however, approximately 40,920 are expected to die from the disease [4]. This emphasizes the need to improve our understanding of BrCa progression to design more effective treatments. Many factors are implicated in BrCa metastasis; however, the precise mechanism involved in the migration of cancer cells to distant organs is unclear [5,6]. 

Chemokines are proinflammatory chemoattractant cytokines that function primarily in leukocyte trafficking and other biological activities, such as development, angiogenesis, and hematopoiesis [7,8,9]. In addition to their role in several pathological conditions, it has become progressively evident that chemokines and their receptors are significant in determining cancer cells’ metastatic destination [1]. CXCL16 is a chemokine that functions in its membrane tethered (insoluble) form and soluble form after proteolytic shedding of the ectodomain [10,11,12,13]. Its trans-membrane form (Tm-CXCL16), on the surface of macrophages, monocytes, and dendritic cells, functions as an adhesion molecule for CXCR6-positive cells [11]. On the other hand, soluble CXCL16 (sCXCL16) is implicated in instigating increased migratory and invasive potential of high CXCR6-expressing cancer cells [14]. CXCL16 is also expressed in various cancers, including prostate, non-small cell lung, colorectal, and breast [10,14,15,16]. It is the conversion of Tm-CXCL16 to sCXCL16 that is crucial for switching to its oncogenic form. However, the exact role of CXCR6 and CXCL16 in BrCa progression [10] or the proteases involved in the release of sCXCL16 is unknown.

ADAMs are a family of proteins involved in proteolytic shedding of extracellular fragments of membrane-bound proteins [17,18,19]. Increased levels of ADAM10 render resistance to trastuzumab [20]. Interestingly, proteolytic shedding of CXC chemokine by ADAM10 is essential for determining cellular migration and proliferation, and cellular fate [12,18]. Therefore, ADAM10 could potentially promote the progression of BrCa by the release of CXCL16 [18]. Here, we show the biological and clinical significance of the ADAM10 and CXCR6/CXCL16 axis on activating molecular pathways involved in BrCa progression.

## 2. Materials and Methods

### 2.1. Tissue Specimens

Tissue microarray (TMA) slides containing breast cancer tissues were procured from Cooperative Human Tissue Network (CHTN), UAB at Birmingham. Retrospectively collected de-identified tissues were used. TMA consisted of 12 stage-II and 63 stage-III BrCa tissue samples. A qualified pathologist confirmed the histopathology and validated the class and tumor grade of each TMA core.

### 2.2. Immunohistochemical Staining and Evaluation of CXCR6/CXCL16 and ADAM10

TMAs were stained for CXCR6, CXCL16, and ADAM10 using methods we have described previously [14,21]. Virtual slides were created with an Aperio ImageScope (Aperio Technologies) to analyze the immunohistochemical staining. True-color digital images of each stained sample were viewed using Aperio ImageScope v.6.25 software. An algorithm for determining the intensity of membrane-specific staining was used to calculate, for each sample, the staining intensity and percent of the target label by digitally analyzing the color intensity. A color markup image for each slide was obtained based on the membrane staining intensity. The output was viewed as determinations of staining intensity ranging from 0–3 to correlate with conventional manual scoring methods (where 0 = negative and 3 = strong staining), and statistical analyses were performed using the mean values.

### 2.3. Cell Culture

The normal mammary epithelial cells (MCF-10A) and BrCa cell lines MCF-7 and MDA-MB-231 were obtained from ATCC. Complete growth medium for MCF10A was prepared by supplementing the base medium (MEBM) with 100 ng/mL cholera toxin provided with the kit (MEGM, Kit Catalog No. CC-3150) procured from Lonza. MCF-7 cells were maintained and cultured in MEM (Hyclone), while MDA-MB-231 were cultured in L-15 containing 10 μg/mL insulin. Culture media for both BrCa cell lines were supplemented with 10% fetal bovine serum (FBS), 1% penicillin and streptomycin, and amphotericin-B. Cultures were grown at 37 °C with 5% CO_2_.

### 2.4. Cancer Signaling Phosphorylation Antibody Array

CXCL16/CXCR6-induced changes in the phosphorylation of various cancer-associated proteins in BrCa cells were assessed using cancer signaling antibody array containing 273 antibodies. These included antibodies against different phosphorylation sites of molecules involved in cancer signaling. Vehicle-treated cells served as the control. Protein lysates were processed and probed onto cancer signaling phospho-antibody microarray slides as per the manufacturer’s protocol (Fullmoon Biosystems Inc., Sunnyvale, CA, USA). Briefly, protein lysates were collected using beads followed by their biotinylation. Biotinylated protein samples were then incubated with the array slides bearing the antibodies, followed by labeling with a dose of 0.5 mg/mL Cy3-Streptavadin as per the manufacturer’s instructions. Slides were then scanned and quantified using an Axon GenePix 4000B microarray scanner (Molecular Devices, Sunnyvale, CA, USA).

The median signal intensity for each antibody was obtained using six replicate spots and was normalized to the median signal of GAPDH. Fold changes in the P/N ratio (phosphorylated/total protein) were calculated by dividing normalized average signal intensities for CXCL16-treated samples by that of controls. The CIMminer platform (https://discover.nci.nih.gov/cimminer/oneMatrix.do (accessed on 29 June 2021)), developed by the Genomics and Bioinformatics Group at the National Cancer Institute, was used to generate a heat map based on the data obtained. Results were divided into datasets and uploaded into Ingenuity Pathways Knowledge Database as text files containing GenBank accession numbers.

### 2.5. Image-Based Analysis of CXCR6 Expression

Briefly, BrCa cells (5 × 10^6^) were harvested and stained with PE-conjugated anti-CXCR6 for 40 min at 4 °C, and the nucleus was stained with DRAQ5™ (Biostatus Ltd., Shepshed, UK). ImageStream was used to acquire images of CXCR6-expressing cells. Analysis was done using the Image Data Exploration and Analysis Software “IDEAS” (Amnis Corp, Seattle, WA, USA).

### 2.6. Cell Migration and Invasion Assay

Migration and invasion of BrCa cells-MCF-7 and MDA-MB-231 were assessed using BD Bio-coat migration or Matrigel invasion chamber systems (BD Biosciences), respectively, as described earlier [16]. Briefly, matrigel inserts were hydrated for 2 h with warm serum-free bicarbonate-based medium (DMEM) in an incubator at 37 °C with 5% CO_2_. After hydration, the media was gently aspirated from the chambers. Next, 800 uL of respective medium containing 2% FBS supplemented with or without CXCL16 (100 ng/mL) were added to the bottom chamber. Then, 10^4^ cancer cells treated with mouse anti-human CXCR6 antibodies (1 µg/mL) or small molecule inhibitors of PI3Kp110α (PI-103, 3 μM), PI3Kp110β (TGX221, 1 μM), and PI3Kp110γ (AS605240, 3 μM), wortmannin (1 μM), Src (SU6656, 5 μM), FAK (PF-573228, 5 μM), or ERK (10 μM) in 400μL of 2% FBS-containing media were added to the top chamber of the inserts and incubated overnight. After incubation, cells on the upper surface of the membrane that did not migrate or invade were removed with a cotton swab. Cells on the bottom surface of the insert were fixed with 100% methanol for 2 min, stained for 30 min in 1% crystal violet, and rinsed twice with distilled water. The membranes were cut and placed on glass slides. Images were captured at 20X magnification, and cells were then counted. The invasion index was calculated by dividing the mean of cells invading through the Matrigel insert membrane by the mean of cells migrating through the control insert membrane multiplied by 100. These assays were repeated three times.

### 2.7. Actin Polymerization

BrCa cells (10^4^ cells/chamber) were cultured on chamber slides and were treated with 100 ng/mL CXCL16 for 10 min with or without pretreatment of anti-CXCR6 (1 ug/mL) or Src (2.5 µM SU5565) or FAK (1 µM PF-573228) or ERK (20 µM PD98059) inhibitors. Then, actin was stained with rhodamine-phalloidin (1.0 ug/mL) for 20 min, and the nucleus was stained with DAPI using the procedure as described in our publication [22]. Images were taken using a confocal microscope.

### 2.8. Statistics

The statistical significance for differences in CXCL16, CXCR6, and ADAM10 expression between groups was analyzed using Mann–Whitney U test. Student’s t-test was used to analyze migration and invasion data. For all tests, *p* values < 0.05 were considered significant. Statistical analyses were performed using Prism software (GraphPad, La Jolla, CA, USA).

## 3. Results

### 3.1. Expression of ADAM10, CXCL16, and CXCR6 in BrCa Tissues and Cell Lines

**Figure 1:**
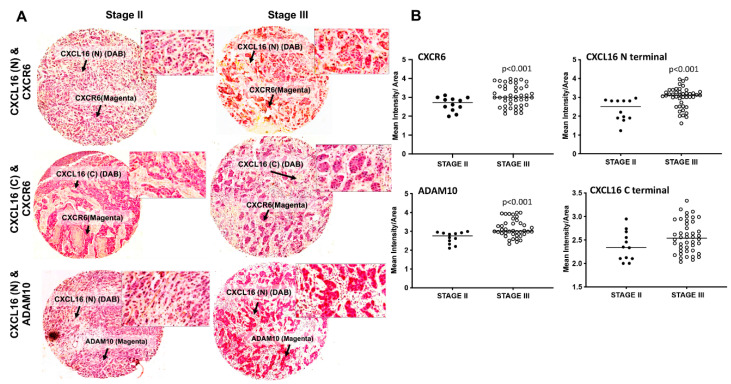
Expression of ADAM10, CXCR6, and CXCL16 in breast cancer tissues. Tissue microarrays consisting of stage-II (*n* = 12) and stage-III (*n* = 63) BrCa tissues were analyzed by immunohistochemistry. Representative images of tissue sections along with TMA spots are shown in Panel (**A**). Brown (DAB) color shows CXCL16 staining, and magenta shows CXCR6 or ADAM10 expression. Quantitation of immunohistochemistry is shown in Panel (**B**). Expression of CXCR6, N-terminal CXCL16, and ADAM 10 was higher in stage-III than stage-II BrCa tissues. There was no significant difference in the levels of C-terminal CXCL16 in BrCa tissues of different stages. The statistical significance for differences in expression between groups was analyzed using the Mann–Whitney U test.

**Figure 2:**
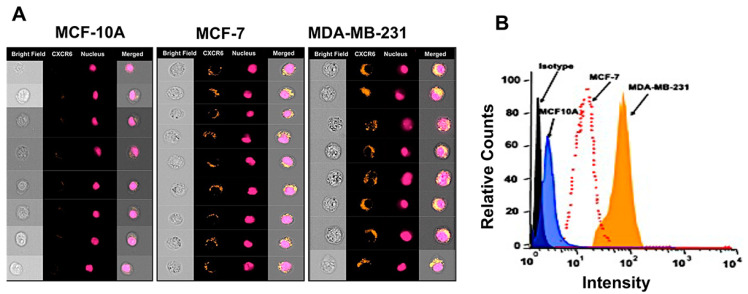
CXCR6 expression in breast cancer cell lines and normal epithelial cell lines. BrCa cells (MCF-7 and MDA-MB-231) and normal mammary epithelial cells (MCF-10A) were stained with PE-conjugated mouse anti-human CXCR6 or mouse IgG2a isotype control antibody. The images of stained cells (10^5^ events) were acquired using an Image stream system. Panel (**A**) shows representative images of few cells. Panel (**B**) shows the analysis after spectral correction using Image Data Exploration and Analysis Software (Amnis, Seattle, WA, USA).

Expression analysis of CXCL16 and ADAM10 in BrCa tissues is important because of the CXCL16-CXCR6 ligand-receptor system in cancer development and the ability of ADAM10 to cleave Tm-CXCL16. Expression levels of CXCR6, as assessed by IHC using BrCa tissue microarray, were higher in advanced BrCa tissues (Figure 1A). The expression of ADAM10 and N-terminal CXCL16 levels were significantly higher in stage-III BrCa tissues than in stage II (Figure 1B). The levels of C-terminal CXCL16 were not considerably different in stage-II versus stage-III BrCa tissues (Figure 1B). BrCa cells were found to express more CXCR6 compared to normal mammary epithelial cells. More tumorigenic MDA-MB-231 cells expressed significantly higher CXCR6 than MCF-7 cells and MCF10A (Figure 2A,B).

### 3.2. Molecular Pathways Activated by the CXCR6/CXCL16 Axis

**Figure 3:**
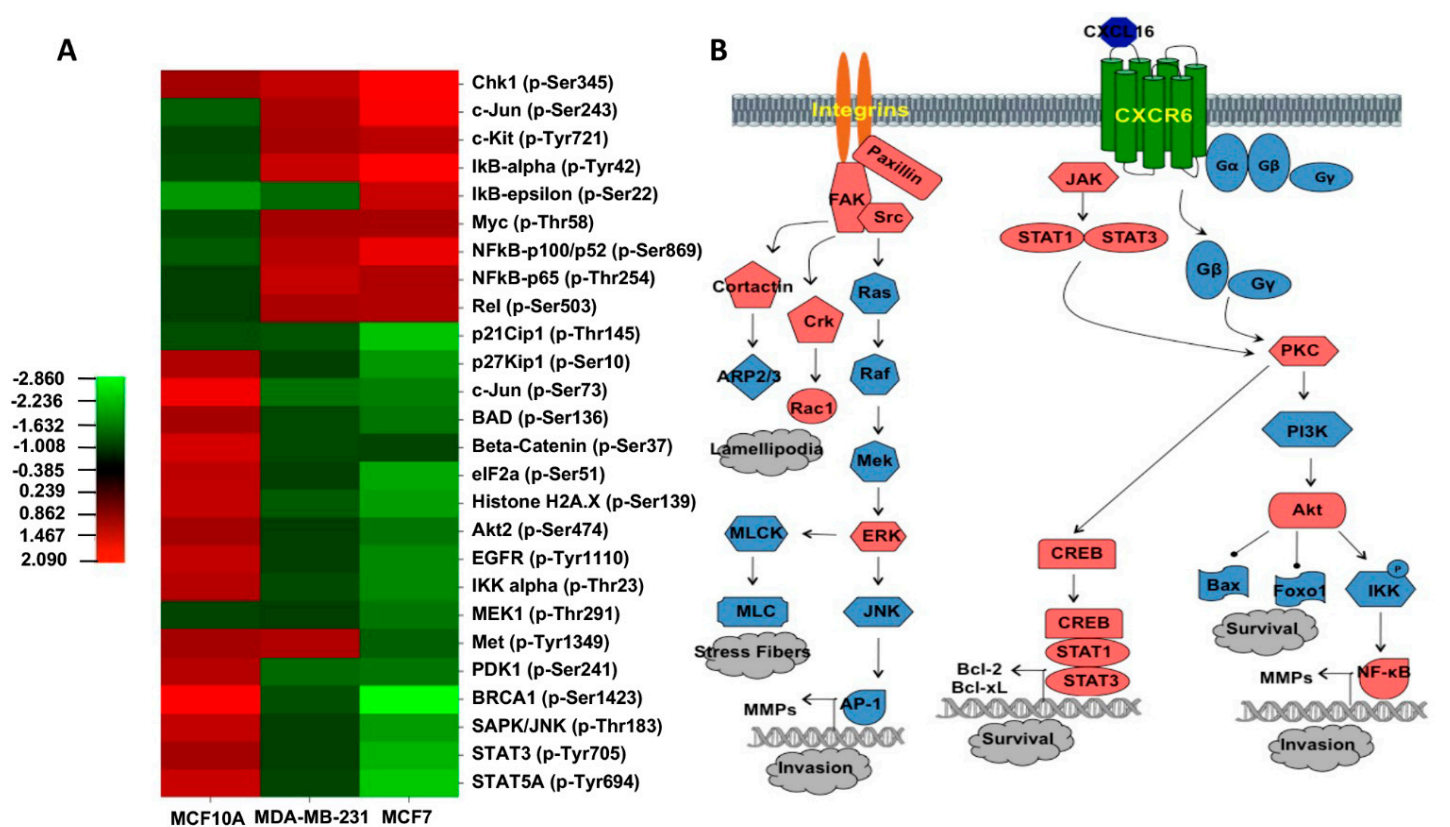
Analysis of CXCR6-mediated phosphorylation of cell signaling molecules using antibody microarray. Phosphorylation-specific antibody array was performed for the qualitative analysis of differentially phosphorylated proteins after treating BrCa cells with CXCL16 (100 ng/mL for 5 min). The BrCa cells treated with vehicle were used as the control. The antibody array consisted of 273 highly specific antibodies against proteins significant for cancer signaling pathways, with β-actin and GAPDH serving as internal positive controls. The median signal intensity for each antibody was obtained using six replicate spots (*n* = 6) and was normalized to the median signal of GAPDH. The heat map shown in Panel (**A**) represents a fold change in the phosphorylation status of specified proteins in CXCL16-treated BrCa cells. Heat map was generated from normalized intensity data using the CIMminer tool. Each cell in the heat map shows the ratio of phosphorylated (P) to non-phosphorylated (N) protein in a treated vs. nontreated sample. Red indicates an increase, while green represents a decrease in phosphorylation signaling molecules. The intensity of the color is dependent on the degree of phosphorylation. Panel (**B**) shows the predicted signaling pathway induced by CXCR6-CXCL16 activation leading to neoplastic progression. Changes in the phosphorylation of signaling molecules obtained from antibody microarray analysis were uploaded into Ingenuity Pathways Knowledge Database as text files containing GenBank accession numbers. The chart shows possible activation (red) of the FAK-Src, PI3K/Akt, and Jak/Stat pathways along with the reorganization of the cytoskeleton, which would increase the potential of BrCa cells to survive and invade.

Upon binding to cell surface receptors, chemokines stimulate intracellular signaling pathways that play a key role in regulating cellular processes to reorganize the actin cytoskeleton [23]. Major mediators of these intracellular signaling pathways are overexpressed in several types of cancers [16] and invasive tumor cells in breast tumors [24]. To understand which of these mediators were activated in CXCR6-expressing BrCa cells when stimulated with CXCL16, we screened 273 different proteins associated with survival, invasion, migration, and rearrangement of the cytoskeleton. Proteins differentially phosphorylated in response to CXCL16 in MDA-MB-231 and MCF-7 compared to MCF10A (Figure 3A) included: Beta-Catenin (Ser37), BRCA1 (Ser1423), Chk1 (Ser345), C-Jun (Ser243 and Ser73), eIF2a (Ser51), Histone H2A.X (Ser139), IKB-alpha (Tyr42), IKK alpha (Thr23), NFkB-p100/p52 (Ser869), NFKB-p65 (Thr254), p21Cip1(Thr145), p27Kip1(Ser10), PDK1(Ser241), SAPK/JNK (Thr183), STAT3 (Tyr705), and STAT5A (Ser780). Of these, phosphorylation of SAPK/JNK (Thr183), c-Jun (Ser73), IKK-alpha (Thr23), STAT3 (Tyr705), and STAT5A (Tyr694) were significantly reduced in both BrCa cells after treatment with CXCL16. Phosphorylation of Chk1 (Ser345), c-Jun (Ser243), IkB-alpha (Tyr42), NFkB-p100/52(Ser869), and NFkB-p65 (Thr254) was significantly increased. Mainly, the signaling molecules of the JNK and NFKB pathways were hyperactivated while checkpoints and apoptosis-inducing signals were reduced.

To further understand which biological pathways related to BrCa were altered by CXCR6/CXCL16 signaling, we fed the antibody microarray data in the Ingenuity Pathways Knowledge Base (IPKB). The analysis showed more neoplasia conducive signaling in BrCa cells than MCF10A after treatment with CXCL16 (Figure 3B). Specifically, Src, FAK, ERK1/2, and PI3K/Akt pathways were activated after the CXCR6 stimulation of BrCa cells. These pathways cumulatively affect the metastatic potential of cells by reorganizing actin polymerization, migratory capacity, and basement membrane invasion [25,26,27,28].

### 3.3. CXCR6 Signaling Promotes BrCa Cell Migration through Src, FAK, and ERK1/2 Pathways and Invasion through the PI3K Pathway

**Figure 4:**
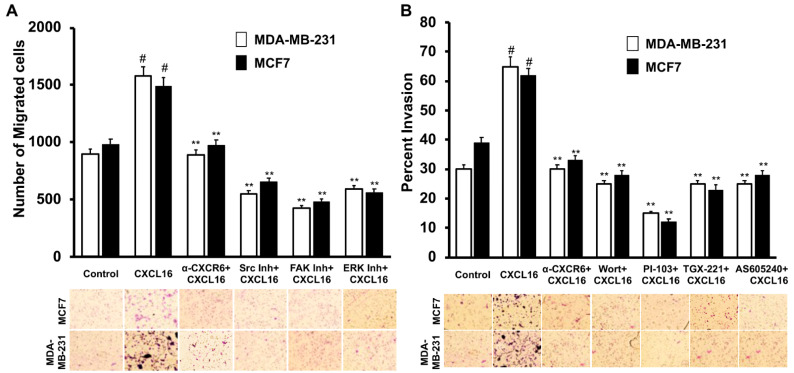
CXCR6 signaling promotes BrCa cell migration and invasion through Src, FAK, ERK1/2, and PI3K/Akt pathways. The addition of chemotactic gradients of CXCL16 resulted in the migration of CXCR6-expressing BrCa cells compared to untreated cells, as shown in Panel (**A**). Open and solid bars represent the results for MDA-MB-231 and MCF-7, respectively. Blocking of the chemokine and receptor interaction with anti-CXCR6 antibody reduced the migration of BrCa cells. Src (SU6656, 5 μM), FAK (PF-573228, 5 μM), and ERK inhibition also significantly reduced the migration of these cells. A significant number of MCF-7 (solid bar) and MDA-MB-231 (open bar) cells also invaded to the lower chamber towards higher CXCL16 in a CXCR6-dependent manner as shown in Panel (**B**). Inhibition of PI3Kp110α (PI-103, 3 μM), PI3Kp110β (TGX221, 1 μM), and PI3Kp110γ (AS605240, 3 μM), wortmannin (1 μM) impaired the CXCL16-led invasion of BrCa cells. Significant differences between groups were analyzed by student’s t-test using mean values obtained from three independent experiments (*n* = 3). For all tests, *p* values < 0.05 were considered significant. #, *p*-value < 0.05 compared to a vehicle-treated group of the corresponding cell line; **, *p*-value < 0.01 compared to the CXCL16 group of the corresponding cell line.

Chemokine receptor signaling plays a crucial role in the metastasis of cancer cells to distant organs. BrCa cells showed ~45% more migratory potential under the chemotactic gradient of CXCL16 than the control cells not subjected to the gradient (Figure 4A). Similarly, more BrCa cells invaded under the CXCL16 gradient than controls with no chemoattractant (Figure 4B).

CXCR6 receptor blockade using anti-CXCR6 antibody reduced CXCL16-induced migration as well as invasion of BrCa cells. With CXCR6 blockade, the migration and invasion of BrCa cells were comparable to control cells not subjected to the CXCL16 gradient (Figure 4A,B).

When BrCa cells were pretreated with ERK, FAK, and Src inhibitors, their migratory potential towards the CXCL16 chemotactic gradient was significantly reduced (Figure 4A). Further, to understand the mechanisms involved in the invasive potential of the BrCa cells, multiple PI3K inhibitors, namely Wortmannin, PI-103 (PI3K 110α inhibitor), TGX-221 (PI3K 110β inhibitor), and AS605240 (PI3Kγ inhibitor), were used. Each of these inhibitors impaired the CXCL16-mediated invasion of MDA-MB-231 and MCF-7 BrCa cells (Figure 4B). Thus, taken together, MAPK/ERK and FAK/PI3K pathways play a significant role in the CXCR6-mediated migration and invasion of BrCa cells.

### 3.4. CXCR6 Signaling Promotes F-Actin Polymerization in BrCa Cells

**Figure 5:**
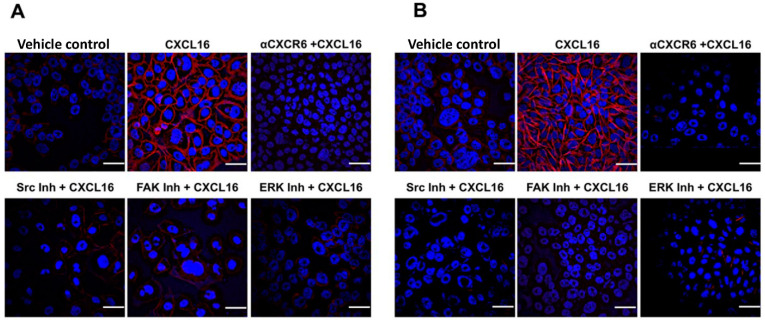
CXCR6 signaling promotes F-actin polymerization in breast cancer cells. Immunofluorescent images show FITC-phalloidin in CXCL16-treated MCF-7 (Panel (**A**)) and MBA-MD-231 (Panel (**B**)) cells. The cells were stained with DAPI to visualize nuclei (blue), and actin was probed with rhodamine-phalloidin (red). Stimulation with CXCL16 increases F-actin polymerization, which was repressed with an anti-CXCR6 antibody. Actin polymerization was significantly reduced by the inhibition of Src, FAK, and ERK1/2. Images with a 20-micron scale bar were captured at 20X magnification and represent three independent experiments (*n* = 3).

BrCa cells treated with CXCL16 showed increased F-actin polymerization, and this actin polymerization was blocked when the activation of CXCR6 by CXCL16 was blocked with anti-CXCR6 **(**Figure 5A,B). To further define the CXCR6/CXCL16 axis-induced signaling involved in actin polymerization, BrCa cells were pretreated with Src, FAK, and ERK inhibitors prior to CXCR6 activation. Reduction in actin polymerization was noted with Src, FAK, and ERK inhibitors, suggesting CXCR6-mediated activation of these signaling molecules is involved in cytoskeletal rearrangement during BrCa cell migration and invasion (Figure 5A,B).

## 4. Discussion

Chemokines are small molecular weight (8–14kDa) chemotactic cytokines that coordinate the migration and positioning of cells by activating the G-protein-coupled chemokine receptors (GPCRs) [29,30]. Aberration in the expression of chemokines and their cognate receptors is associated with several types of cancer development and metastasis [8]. The CXCR4/CXCL12 chemokine axis’s role has already been established in BrCa metastasis [31,32]. Muller et al. (2001) reported that CXCR4/CXCL12 axis blockade suppresses BrCa metastasis to the lung [31]. Expression of CXCR6 and CXCL16, comprising another significant chemokine axis, is evaluated in several cancers, such as renal [33], rectal [34], pancreatic ductal adenocarcinoma [35], nasopharyngeal carcinoma, and melanoma [36]. We have shown the significance of CXCR6 and CXCL16 in the lung and prostate in our previous studies [14,16]. The current study shows upregulation of the receptor and ligand in advanced BrCa tissues and that stimulation of cellular CXCR6 with CXCL16 induces cytoskeleton remodeling and increases migration and invasion of BrCa cells. The effect is CXCR6 dependent. These results substantiate that increased expression of CXCR6 and CXCL16 leading to hyperactivation of the ensuing signaling is associated with BrCa progression.

Newly synthesized CXCL16 is transported to the cell surface and suppresses the tumor proliferation while tethered to the membrane [36,37]. Certain conditions induce proteolytic cleavage of the Tm-CXCL16, causing the release of the N-terminal fragment, the oncogenic (sCXCL16) form [36,37], to the outside of the cell [38]. ADAM10 has often been proposed to cleave CXCL16 from the cell membrane [38]. We observed elevated expression levels of ADAM10 and N-terminal CXCL16 in stage III versus stage II BrCa tissues. Consistent with our results, Gaida et al. (2010) also observed an upregulation of ADAM10 in pancreatic cancer and demonstrated its involvement in cancer cell migration and invasion of cells [39]. ADAM10 also increases the migration potential of cancer cells through the Notch 1 signaling pathway [40]. It could also promote cell migration via αVβ5 integrin in an ERK- and FAK-dependent manner by cleaving L1CAM [41]. Similar to this, the role of CXCL16 in supporting cell migration via αVβ3 integrin in prostate cancer cells is established [16]. We observed that BrCa cells expressing CXCR6 selectively migrated and invaded towards CXCL16 in a CXCR6-dependent manner. Thus, this axis plays an equally important role in BrCa progression by promoting the metastatic capacity like in other malignancies [14,16]. The migration and invasion-promoting effect of the CXCR6/CXCL16 chemokine axis depends on Src, FAK, and ERK1/2 activity in BrCa cells since FAK and Src pathway suppression decreased the migratory and invasion potential. These intracellular tyrosine kinases (FAK, PI3K/Akt, ERK1/2, and Src) play a significant role in cancer metastasis either independently or in conjunction with other pathways that affect cell adhesion, migration, invasion, and proliferation [6,42,43,44,45,46,47].

BrCa cells stimulated with CXCL16 showed changes in the phosphorylation status of molecules key to cancer signaling. CXCL16 increased IkB-alpha Tyr42 phosphorylation that would release sequestered NFkB; CKII phosphorylates this site under oxidative stress conditions. Surprisingly, however, IKK-alpha, a kinase that phosphorylates IkB-alpha to release NFkB, was mostly in its dephosphorylated, less active state after CXCR6 activation. These, together with phosphorylation of NFkB polypeptides, including Ser869 and Thr254, imply that CXCR6 activation in BrCa cells would enhance NFkB processing. Intriguingly, L1CAM (product of ADAM activity) facilitates NFkB signaling, promoting cell motility in colon cancer [48]. Therefore, based on our data and previous findings, it is very likely that ADAM10-induced CXCL16 cleavage could trigger classical NFkB signaling promoting stem cell renewal, epithelial-mesenchymal transition (EMT), and metastasis via L1CAM–Ezrin-integrin signaling.

BrCa cells treated with CXCL16 also showed decreased GSK3B-mediated phosphorylation of B catenin Ser37, which suggests increased B-catenin stability, which in turn would support survival, EMT, and metastasis. Additionally, Wnt/B-catenin and NFkB signaling can influence each other at multiple levels to promote oncogenesis.

Besides, c-Jun Ser243 phosphorylation, representing Ser/Thr phosphatase calcineurin activity, increased in BrCa cells in response to CXCR6 activation. This increase is associated with decreased protein stability and increased tumorigenic ability [49]. Ser73 phosphorylation of c-Jun mediated by SAPK/JNK mediates transcription-dependent apoptotic signaling in neurons [50]. Treatment with CXCL16 reduced this Ser phosphorylation in BrCa cells, which suggests decreased SAPK/JNK activity that is evident from the reduced phospho-Thr183 levels as well. Thus, CXCL16 affects c-Jun signaling in BrCa cells. However, its effect on proliferation and survival needs to be further investigated.

In addition to enhancing the pro-survival and EMT signaling, CXCL16 also negatively affected apoptotic signaling and cell cycle checkpoints. A significant reduction in phosphorylation of Ser1423 in both cell types and Ser1524 residues in MCF-7 compared to normal epithelial cells indicates reduced caspase-3-mediated apoptosis in BrCa cells. CXCL16 also reduced Ser216 phosphorylation of CDC25C that could lead to increased cell division. Thus, stimulation with CXCL16 could suppress apoptotic signaling, facilitate overriding cell cycle checkpoints, promote EMT, and ultimately confer a survival benefit to BrCa cells via its effects on Akt, NFkB stability and processing, BRCA, and the SAPK/JNK pathway.

One of the first events of the cell migration in response to chemoattractant is cell polarization mediated by actin polymerization [6,51,52]. FAK plays a vital role in focal adhesions, and it activates other molecules involved in cell movements, such as p130cas, crk, and paxillin [53]. The FAK inhibitor effectively reduced BrCa (MCF-7 and MDA-MB-231) cell migration and invasion towards CXCL16 gradients in our study. Similarly, a decrease in cell migration and invasion was noted after inhibiting Src in BrCa cells. Further, inhibition of FAK and Src also reduced CXCL16-induced F-actin polymerization. Thus, our results show that CXCR6/CXCL16 signaling induces migration and invasion of BrCa cells mediated through FAK- and Src-triggered F-actin polymerization.

The ERK pathway is one of the MAPK pathways that regulate cell proliferation; its deregulation is linked to several human cancers [54]. Studies have shown that activation of ERK1/2 triggers a cascade of Rho activation-inhibition of cofilin to induce F-actin polymerization [10]. It controls the rate and actin polymerization timing [6,55]. Interestingly, chemokines are known to activate the MAPK/ERK pathway [8,56]. Stimulation with CXCR4/CCR7 ligands activates the ERK1/2 pathway in metastatic BrCa cells but not in non-metastatic cells [7]. Using ERK1/2-specific inhibitor, we demonstrated that the CXCR6-CXCL16 axis promotes migration via the MAPK/ERK pathway, which induces F-actin polymerization. We did not deduce the exact mechanism; however, our results show that CXCR6-stimulated migration of BrCa cells involves ERK-dependent F-actin polymerization.

In conclusion, our study provides evidence that the CXCR6-CXCL16 axis promotes F-actin polymerization, eventually enhancing the migration and invasive potential of BrCa cells by the Src, FAK, and ERK1/2 pathways. The CXCR6-CXCL16 axis also activates signaling pathways supporting cell survival, apoptosis, and EMT in BrCa cells. In addition to this, our study shows higher ADAM10 expression in advanced BrCa tissues. ADAM10 may be significant in promoting BrCa progression by proteolytically releasing sCXCL16 and activating oncogenic CXCR6 signaling. Although further studies are required to elucidate the function of ADAM10 in BrCa progression, our observations imply that controlling the release of CXCL16 by targeting ADAM10 could be an effective way to treat advanced BrCa.

## Data Availability

This study did not utilize or reported any data base. However, we will be happy to share additional information regarding the antibody microarray data submitted as supplementary material, if requested.

## Supplementary Materials

The following are available online at https://www.mdpi.com/article/10.3390/cancers13143568/s1.
